# Severe anorexia nervosa, co-occurring major depressive disorder and electroconvulsive therapy as maintenance treatment: a case report

**DOI:** 10.1186/1757-1626-2-9362

**Published:** 2009-12-21

**Authors:** Outi Poutanen, Kaija Huuhka, Kaisa Perko

**Affiliations:** 1 Department of Psychiatry, Tampere University Hospital, 33380 Pitkaniemi, Finland

## Abstract

**Introduction:**

It is difficult to treat patients who, in addition to having severe anorexia nervosa, also have severe symptoms of major depressive disorder and a tendency for impulsive acting out behaviour. Our case report considers the feasibility of maintenance electroconvulsive therapy in such complicated cases.

**Case presentation:**

This is a case report of a woman with anorexia nervosa and co-morbid severe major depressive disorder who was treated with electroconvulsive therapy as a maintenance treatment.

The maintenance electroconvulsive therapy was conducted without immediate complications. It had a positive effect on the patient's depressive symptoms and lability and her general wellbeing, although some cognitive deficits were observed.

**Conclusion:**

The maintenance electroconvulsive therapy seemed to support recovery in a case of refractory anorexia nervosa and a tendency for labile mood. The symptoms of co-occurring major depressive disorder were partly relieved and maintenance electroconvulsive therapy had some positive effect on weight gain.

## Introduction

Psychiatric co-morbidity in eating disorders is high. Affective disorders co-exist most frequently with eating disorders [1,2]. There is no consensus on treatment guidelines in eating disorders [3], and the role of antidepressants (AD) is not clear [4], although in case of co-existing major depressive disorder (MDD) using AD is obvious. Electroconvulsive therapy (ECT) has been among the treatment choices for eating disorders but studies on its effectiveness are only case reports [5-7]. Maintenance ECT (mECT) has a role in the treatment of refractory MDD, although some memory deficits have been reported [8]. The association between cognitive decline and mECT seems to be non-significant [9].

We have a patient with severe anorexia nervosa whose concurrent MDD was successfully treated with mECT.

## Case presentation

The long-term treatment period of a Finnish, ethnically Caucasian, 21-year-old high school graduate and shop assistant, started when she was admitted to the acute ward of the local psychiatric hospital in August 2007 with a diagnosis of anorexia nervosa according to the criteria of the ICD-10. Her eating disorder had started in 2001, when she was 13 and began to exercise and avoid fattening food. Her weight dropped from 78 kg to 49 kg (her height is 178 cm) in a few months. At that time her parents were having severe marital problems. However, the parents did not separate or divorce. At the same time Ms. P.M. had started high school in a boarding school about 50 km away from her home, which was an exceptional choice as boarding schools are a rarity in Finland.

During the first months of her eating disorder she had psychiatric outpatient treatment, but in summer 2002 her condition deteriorated as she started bulimic behaviour and vomiting, and also had symptoms of depression (depressive mood, loss of pleasure, low energy, insomnia). In September 2003 she was admitted to the Department of Adolescence Psychiatry, Tampere University Hospital and hospitalized for two months. During her hospital treatment she had no psychotropic medication. Her relationship to her parents was found to be exceptionally close and she had only one friend of her own age. There were many unsolved family problems but abuse was not among them. However, the eating disorder had brought the parents closer to each other, which pleased the patient.

The patient subsequently resumed psychiatric outpatient treatment until spring 2005. The outpatient treatment consisted of visits to a psychologist with CBT training once a week, psychotropic medication (fluoxetine 40-60 mg/day), and nutritional advising. She was able to complete her high school studies with good grades in May 2005. She then got a job as a shop assistant. She moved away from her parents' home with her only (female) friend as a room-mate. She also began occasionally dating a boyfriend of her own age and the relationship was a good one. In summer 2007 her room-mate moved to another town to work. Soon after that the patient's eating disorder became more severe and she had amenorrhoea. She also began to mutilate herself with a knife and to drink quite a lot of alcohol (cider). She was admitted to the acute ward of the local psychiatric hospital in August 2007. At that time her weight was 56.2 kg and Body Mass Index (BMI) 17.7. The patient's hospital treatment was then two months. After that there was an outpatient period of seven weeks, a new hospital treatment of one month, and again an outpatient treatment of six weeks. Poor compliance was the biggest problem in this outpatient treatment.

Since the beginning of April 2008 she has been almost continuously hospitalised for more than a year (there was a short period of outpatient treatment in August 2008). She began her psychiatric inpatient treatment again in the local hospital. At the beginning of summer 2008 her weight was 40.0 kg and BMI 12.6. At that time she was admitted for two weeks for rapid nasogastric refeeding on the medical ward of the local hospital. Her weight rose to 49.6 kg in only two weeks, making her mood more and more anxious. Thereafter she was admitted to the psychiatric ward of the University Hospital, again for four days on the medical ward of the University Hospital, and then back to the psychiatric ward. Besides anorexia nervosa (F50.0), major depressive disorder (MDD; F32.2) was also diagnosed according to the criteria of the ICD-10. Her symptoms of depression were: lowering of mood, reduction of energy, decrease in activity, reduced capacity for enjoyment and interest, hopelessness, appetite diminished, feelings of worthlessness, agitation and suicidal acts. She had delusional thoughts about her body shape and weight. She totally refused to eat and regarded even water as a fattening foodstuff. She tried to vomit aggressively and exercise physically all the time. Mechanical restraints, nasogastric refeeding, and constant observation 24 hours a day were provided. She nevertheless managed to abuse herself by hysterical cutting and vomiting. At that time her medication included fluoxetine (60 mg/day) and olanzapine (30 mg/day) and also lorazepam (2 mg/day) before main meals. Later on fluoxetine was supplied in a combination of venlafaxine (150 mg/day) and mirtazapine (60 mg/day). Because the symptoms of MDD were not relieved with AD treatment, ECT was used. ECT was administered three times a week. Anaesthesia was induced with methohexital and muscle relaxation with succinylcholine. Physiological monitoring included pulse oximetry, blood pressure, ECG, four channel EEG and EMG. She had ten bifrontotemporal ECT sessions in July 2008. The psychotropic medication was continued during the ECT treatment. There were no anaesthetic complications. The respective Beck Depression Inventory (BDI) [10] scores were 52 and 35 before and after the ECT treatment, meaning that her depression was not totally relieved. Clinically, the patient was satisfied with the outcome of the ECT, her behaviour continued to be self-destructive but her mood was better, she was more active, no longer hopeless, no longer delusional, and her appetite was no longer impaired. BMI had risen only slightly from 15.0 to 15.3. She continued exercising but the vomiting had diminished.

In August 2008 she first spent three weeks on the psychiatric ward of the local hospital and then returned to the psychiatric ward of the University Hospital with BMI only 13.8. She had started to exercise aggressively again. At the beginning of September 2008 her BMI was only 12.2. Two short treatment periods of nasogastric refeeding on the medical ward were initiated. She then requested a new series of ECT. Before the ECT her BDI was 35, her Montgomery-Åsberg Depression Rating Scale (MADRS) [11], score 28 and BMI 14.2. After 12 bifrontotemporal ECT treatments BMI had risen slightly to 14.6, her mood was slightly better according to MADRS (scoring 24) and she began to smile, but she was acting out (lying and stealing) and the self-rated BDI was higher than it was before the ECT: 43. Vomiting and exercising had diminished quite a lot. In October 2008 she was transferred to a psychiatric ward specialized in treating refractory patients. At that time a decision was taken on mECT: bifrontotemporal ECT once a week or once in two weeks. The medication was again supplied: quetiapine (600-800 mg/day) was used instead of olanzapine and fluoxetine (60-70 mg/day) instead of other ADs. She had creative art therapy and physiotherapy in order to improve her body image of which physiotherapy was of some help by teaching the patient to relax. In December 2008 she was allowed to spend some days at (her parents') home. By the end of 2008 BMI was16.4 and she had no symptoms of depression. During the first weeks of 2009 mECT was only given once in 4 weeks. Very soon she became anxious again and started exercising, stealing sweets, binge eating, vomiting with the help of various hoses, and evacuating her rectum. Her mood became low and she was tired and hopeless. In the middle of February 2009 the frequency of mECT was then temporarily increased to twice a week for two months. Her mood improved and she was active and optimistic. Her weight remained at 50-51 kg (BMI about 16) until the beginning of April. The most aggressive exercising and vomiting faded away. Then quetiapine was changed to aripiprazole (20-30 mg/day), and at the same time the frequency of mECT was changed to once in 2 weeks. A new type of therapy was also initiated: the patient was given more responsibility for her own behaviour and the use of strict weight limits in order to obtain new benefits was abandoned. Her mood remained in balance and the acting out behaviour (stealing, lying, hysterical cutting) declined, but her weight dropped rapidly from 51 kg to 46 kg (BMI 14.5) in only eight weeks.

Ms. P.M. underwent a series of 10 ECT in the summer, a series of 12 ECT in the autumn, and a total of 23 mECTs during the winter and spring 2008-9. In order to control for possible side-effects of the mECT, a magnetic resonance image (MRI) of her head was taken and a psychologist's assessment was conducted in May 2009. The MRI was normal. Maintenance ECT was discontinued in order to get a psychologist's assessment of memory functions during a four-week time free of ECT. Soon depressive symptoms again became prominent: low mood, reduction in energy, difficulties in concentrating, but no hopelessness or suicidality. BMI remained low at 14.5. However, according to the psychologist's assessment in May 2009, the patient's cognitive capacity had been impaired during the last 21 months. A shorter psychological assessment had been conducted in August 2007 and at that time the patient's cognitive skills were clearly better (IQ was 116 in August 2007 and 102 in May 2009). Moreover, her memory functions three weeks after the latest ECT yielded a very uneven memory profile, indicating difficulties in verbal memory (word lists) possibly due to fluctuating attention. The psychologist's assessment even suggested that the patient's semantic memory might have deteriorated. As we did not want to expose her to possible difficulties with memory the idea of more mECT was abandoned.

She has now been 10 weeks without mECT. Her regular medication has been aripiprazole 30 mg/day, fluoxetine 60 mg/day and lorazepam 1 mg twice a day before main meals. At first her mood became depressive, but after 5-6 weeks she was no longer depressive but only feeling down once in a while. Her BMI has slightly risen from 14.8 to 15.3.

## Discussion

Contraindications for ECT in general and in eating disorders are few: Disturbances in electrolytes may elevate the risk of anaesthesiologic complications. Low body weight is not as such a contraindication for ECT [12]. The role of mECT in the treatment of recurrent depression is not consistent [8] and there are no studies about its effectiveness in eating disorders. In our case, mECT seemed to have a stabilizing effect on the patient's mood in a situation including not only anorectic and bulimic behaviour but also MDD and multiple acting out. A great benefit of the ECT procedure was that the patient herself and her family were strongly in favour of ECT and also of mECT.

The most common adverse effects of ECT are memory disturbances consisting of both anterograde and retrograde amnesia [13]. Anterograde memory defect usually disappears in a few days or weeks after completion of the ECT course [12]. In objective measures retrograde memory loss has been found to disappear 6 months post treatment [14]. Difficulties with verbal and semantic memory are not the most typical consequences of ECT but some evidence of deteriorating verbal memory following ECT has been reported [15]. It is possible that in this case impaired cognitive capacity and difficulties with verbal memory were not consequences of mECT but a result of a long-term treatment period on a ward for seriously mentally ill patients (consequences of institutionalization), or they were consequences of MDD, or consequences of malnutrition. It is also possible that the patient had early co-morbid symptoms of some other psychiatric disorder e.g. a functional psychosis.

Our patient had many kinds of treatments. The role of psychotropic medication, creative art therapy, physiotherapy or clinical therapeutic principles was not under observation here. But for instance when the patient was given more responsibility for her own behaviour in April, it was clearly too much for her. In spite of her mood being in better balance her weight dropped rapidly.

## Conclusion

ECT seemed to have a stabilizing effect on the mood and some positive effect on weight gain. The changes in symptoms were transient with ECT but slightly more stable with mECT with a frequency of at least once in two weeks. Altogether we suggest that ECT is a treatment option when a patient has an eating disorder and co-morbid severe MDD. As maintenance treatment mECT seemed to support recovery in a case of refractory anorexia nervosa and a tendency for labile mood. The risk of memory dysfunctions after mECT, however, requires further research.

## Competing interests

The authors declare that they have no competing interests.

## Authors' contributions

OP was clinically in charge of the patient most of the time during this long-term treatment period. OP performed the literature search on the subject, analysed the data, and drafted the manuscript. KH was in charge of the ECT procedure, drafted the parts of the manuscript concerning ECT, and revised the manuscript critically for important scientific content. KP assessed the patient psychologically, drafted the parts of the manuscript concerning cognitive dysfunction and psychological assessment, and revised it critically for important scientific content. All authors have read and approved the final manuscript.

## Consent

Written informed consent was obtained from the patient for the publication of this case report. A copy of the written consent is available for review by the Editor-in-Chief of this journal.
